# Intelligent Decision Support for Transcatheter Aortic Valve Replacement: Machine Learning Spans From Anatomical Assessment to Dynamic Risk Modeling

**DOI:** 10.31083/RCM44364

**Published:** 2026-03-20

**Authors:** Xinjie Hu, Peiling Xie, Ying Li

**Affiliations:** ^1^Department of Digital Medicine, College of Biomedical Engineering and Medical Imaging, Army Medical University (Third Military Medical University), 400038 Chongqing, China

**Keywords:** machine learning, transcatheter aortic valve replacement, anatomical assessment, risk prediction

## Abstract

This study aimed to investigate the application of machine learning (ML) in transcatheter aortic valve replacement (TAVR) and to demonstrate that, owing to the unique strengths of ML, this field outperforms conventional approaches in both preoperative assessment and postoperative prediction of TAVR. Nonetheless, TAVR is the preferred treatment option for medium- and high-risk patients with aortic stenosis, a common valvular disease, because of the associated minimally invasive nature and rapid recovery. However, challenges remain in preoperative evaluation and in predicting postoperative complications. Thus, ML technology offers innovative solutions for these challenges. This study provides an overview of current ML applications in TAVR and evaluates the associated benefits in measuring preoperative anatomical parameters and predicting postoperative complications. Indeed, the superiority of ML models for preoperative planning can be assessed by comparing ML model-derived data with measurements from senior and junior observers across various aortic root anatomical parameters. Additionally, this review discusses the challenges of applying ML in TAVR, including data acquisition, privacy protection, and model generalizability. The ongoing advancement of artificial intelligence (AI) technologies, particularly the integration of explainable AI and federated learning, is expected to enhance the accuracy and personalization of preoperative planning and postoperative prediction for TAVR. This progress will facilitate broader application of these technologies, ultimately benefiting a wider patient population.

## 1. Introduction

Aortic stenosis is the most common valve disease worldwide, and its incidence continues to increase with the aging population [1]. Although Surgical Aortic Valve Replacement (SAVR) remains the gold standard, transcatheter aortic valve replacement (TAVR) offers lower invasiveness, faster recovery, and equivalent long-term outcomes; therefore, its use has shifted from high-risk to low-risk and younger patients [2, 3, 4, 5]. The trade-off is a technically demanding procedure conducted in a confined operative field, carrying specific risks of coronary obstruction or paravalvular leakage, which must be anticipated on a patient-by-patient basis. Durability concerns add further complexity, as younger recipients often require future valve-in-valve or redo-TAVR, where even millimeters of miscalculation can raise residual gradients and precipitate severe prosthesis–patient mismatches [6, 7]. Therefore, accurate preoperative sizing, lifelong surveillance, and reliable prediction of early complications exceed the traditional risk scores. The machine learning (ML) integration of imaging, engineering, and clinical data has become essential for optimizing the entire TAVR pathway. This study aimed to investigate the application of ML in TAVR and demonstrate that ML outperforms conventional approaches in both preoperative assessment and postoperative prediction.

## 2. Challenges in TAVR Precise Evaluation and Postoperative Prediction

TAVR requires rigorous preoperative evaluation to enable personalized interventional planning and improve clinical outcomes; however, current methods are complex and challenging.

### 2.1 Precise Anatomical Quantification Remains Challenging

The success of TAVR depends on precise aortic annulus assessment. The annulus diameter changes with cardiac contraction and relaxation; hence, static measurement is insufficient [8]. In practice, the systolic diameter is larger; therefore, it is usually measured [9]. However, some studies have indicated that the maximum annulus diameter may occur during diastole; therefore, guidelines recommend capturing data for the entire cardiac cycle [10].

Coronary artery obstruction is a common and severe complication of TAVR. Accurate measurement of anatomical parameters, such as coronary artery ostium position, height, relation to valve structure, leaflet length, and calcification extent, combined with hemodynamic modeling, can predict the risk of coronary artery obstruction [11]. In practice, the coronary artery ostium position varies among individuals and is influenced by pathological factors, such as calcification and valve disease, as well as dynamic changes [12]. Moreover, valve implantation-induced coronary artery displacement poses challenges in predicting the risk of coronary obstruction through anatomical parameter measurements.

### 2.2 Valve Sizing and Implantation Decision-Making Remain Difficult

Valve size selection models can be built based on precise anatomical parameter measurements to enhance valve selection accuracy and interventional success. Various measurement strategies for annulus size selection currently exist, including those based on annulus size, balloon angioplasty results, and supraannular structure measurements. Each method has its own advantages and disadvantages, and no unified standards exist [13]. In some cases, the aortic annulus may be in the critical zone [14]. For these patients, whose annulus size is near the boundary of two differently sized valves, clinicians must consider other factors (e.g., calcification extent, coronary artery ostium position, and sinotubular junction size) when deciding between a larger or smaller valve. This increases the complexity of valve size selection [15].

### 2.3 Challenges at the Convergence of ML and Multimodal Imaging

Three-dimensional echocardiography, cardiac computed tomography (CT), and cardiovascular magnetic resonance imaging (MRI) provide the resolution required to grade aortic stenosis, regurgitation, and mitral and tricuspid diseases [16]. Manual contouring is labor-intensive and operator-dependent. ML segments leaflets, annuli, and surrounding structures in real time, color-mapping calcific deposits or jet trajectory. In stenosis, the targets are calcification and root geometry, whereas in regurgitation, the dynamic jet volume and chamber impingement are tracked across four-dimensional datasets [17].

Precise anatomical parameter measurement and valve size selection both affect valve implantation depth. Excessively deep implantation may cause complications such as conduction block. Computer modeling can simulate the valve implantation-induced mechanical behavior, predict the stress distribution and deformation at different depths, and determine the optimal implantation depth [11]. However, this is limited by factors such as pathological changes and dynamic aortic variation measurements.

Beyond the challenging preoperative evaluation, regular postoperative follow-up and monitoring are needed, and rely on accurate postoperative complication prediction, which is another challenge. TAVR complications include cardiac conduction abnormalities, stroke, local vascular complications, contrast-induced nephropathy, heart failure, infection, and pericardial effusion/tamponade [18]. These complications can result in organ ischemia, severe injury, and even death. In addition, as TAVR is increasingly performed in low-risk populations and post-TAVR complication rates rise, patients are placing more emphasis on assessing postoperative mortality and complication risks. Accurate prediction of post-TAVR clinical outcomes is vital for identifying high-risk patients, ensuring robust perioperative planning, and facilitating informed consent.

Traditional cardiac implantation risk prediction scores, such as the EuroSCORE I and II and the Society of Thoracic Surgeons Predicted Risk of Mortality (STS-PRoM), are widely used in cardiac implantation. These scores offer valuable preoperative risk assessment references and have demonstrated accuracy and reliability in predicting mortality and complications in traditional cardiac implantation [19, 20, 21, 22, 23]. However, these scoring systems have limitations when applied to TAVR. Patients undergoing TAVR often have unique clinical features: they are typically older, have multiple comorbidities, and face unique risks associated with minimally invasive implantation. Based on data from traditional cardiac implantation, these scoring systems fail to adequately account for TAVR-specific risks; thus, they are inaccurate and unsatisfactory in predicting post-TAVR mortality and complications.

In recent years, TAVR-specific risk scores, such as the Observant, France 2, Core Valve, and TAVI2 scores, have been developed. These scoring systems, derived from unique clinical and interventional data of patients undergoing TAVR, accurately assess post-TAVR risks. These scoring systems target the unique risks of patients undergoing TAVR (old age, multiple comorbidities, and minimally invasive implantation-related risks), making them more suitable for pre-TAVR risk assessment compared to traditional cardiac implantation risk prediction scores [24, 25, 26]. However, most scoring systems are generated using traditional statistical methods (e.g., logistic regression). These methods assume linear variable relationships and rely on stepwise variable selection, which limits their ability to handle complex multimodal clinical data. Additionally, they are insufficient for individualized assessment, timely data updates, and adequate external validation, rendering their accuracy in predicting postoperative mortality and complications inadequate for high-precision clinical decision-making [26]. Table 1 presents the common data types encountered in clinical practice. The complexities of these data types are determined by the specific scenarios in which they are applied.

**Table 1.** **Data types, complexity, and application scenarios in TAVR quantitative assessment and risk prediction**.

Data type	Complexity	Application scenario
Clinical feature data	These data may have complex inter-relationships, including multidimensional patient information, such as age, sex, and comorbidities.	Enabling determination of patient suitability for TAVR by assessing major risk factors, and assisting in initial risk stratification and long-term prognosis evaluation.
Intraoperative monitoring data	These data are real-time, continuous, and dynamic, such as hemodynamic parameters and ECG monitoring data.	Evaluating immediate postoperative effects, classifying key indicators, capturing dynamic data changes, and predicting potential complications or interventional strategy adjustments.
Imaging data	These data have a large volume and high dimensionality, requiring professional analysis techniques and tools, including CT and ultrasound.	Analyzing preoperative imaging data, automatically identifying structural parameters, measuring key parameters to support interventional planning, and enhancing preoperative imaging analysis to expand training datasets.
Text data	These are unstructured data requiring natural language processing techniques to be transformed into analyzable forms, such as interventional records and follow-up reports.	Processing text information in patient medical records, extracting valuable clinical features, assisting in preoperative risk assessment, and interventional planning.

CT, computed tomography; ECG, electrocardiogram; TAVR, transcatheter aortic valve replacement.

**Fig. 1.**
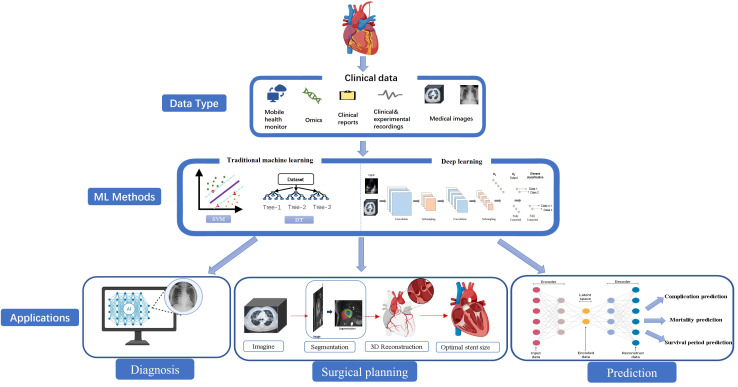
**A framework for the application of machine learning in clinical medical data**. ML, machine learning; SVM, support vector machine; DT, decision tree; AI, artificial intelligence. The figures are created by the authors themselves, with some graphic elements sourced from BioRender.

With the rapid advancement of artificial intelligence (AI), ML has emerged as a powerful tool capable of automatically learning patterns from large volumes of complex data, thereby facilitating efficient and accurate predictions and decision-making. This technology can significantly enhance model accuracy and generalizability, placing it at the forefront of predictive analytics [27, 28, 29, 30]. ML involves the accurate extraction of features from a clinical medical environment and the establishment of models for making informed decisions. However, certain features, such as the geometric characteristics of aortic sinus replication and the spatial distribution of pathological information, are challenging to quantify. Deep learning (DL), a crucial subset of ML, excels in handling high-dimensional nonlinear data, which traditional methods, such as adaptive feature extraction from medical images, struggle with. It can also accurately segment anatomical structures from images for 3D reconstruction and quantitative analysis. Thus, ML is particularly well-suited for tasks involving multimodal information-assisted diagnosis, precise interventional planning, and prediction of postoperative complications. Fig. 1 illustrates the common methods and application frameworks of ML in clinical medical data.

Data associated with TAVR are voluminous and complex, and encompass both structured and unstructured data. These data involve different modalities, various data distribution ranges, and distinct spatial dimensions. ML can effectively address these challenges owing to its high efficiency, accuracy, and adaptability, and has been extensively applied in procedures such as TAVR. Fig. 1 outlines the data ecosystem and application framework of ML in the TAVR care pathway. Beginning with medical images, such as CT and echocardiography, ML or DL performs anatomical segmentation and 3D reconstruction to create high-quality datasets. Based on these datasets, ML models can quantitatively analyze key anatomical parameters to support clinical decisions, including valve selection, interventional planning, and complication prediction, covering the entire chain from preoperative diagnosis to intraoperative guidance and postoperative risk assessment.

## 3. Overview of ML

### 3.1 Common ML Methods

Conventional statistical approaches have limitations in processing high-dimensional datasets and capturing complex nonlinear relationships. However, several problems, such as complex interactions and nonlinearity, are involved in TAVR applications. ML algorithms can address these challenges and handle data complexity effectively. Research has shown that ML can analyze large amounts of complex data, automatically uncover hidden patterns and correlations, and build more accurate predictive models.

As the TAVR-eligible population grows, significant differences have been observed in patient clinical characteristics, risk factors, and prognoses. ML can integrate multidimensional patient data, such as medical history, imaging, and laboratory tests, to create personalized treatment plans. Additionally, it helps clinicians evaluate interventional risks and benefits, thereby aiding in the selection of the most suitable treatment approach.

Table 2 (Ref. [31, 32, 33, 34, 35, 36, 37, 38, 39, 40]) compares common ML and DL algorithms, including their principles, applicable data types, strengths, limitations, and potential applications in the TAVR field.

**Table 2.** **Comparison of different machine learning and deep learning algorithms and their potential applications in TAVR quantitative assessment and risk prediction**.

Algorithm name	Algorithm type	Principle	Data types	Advantages	Limitations	Application scenarios in TAVR
Artificial Neural Network [31]	Machine Learning - Supervised Learning	Simulates human brain neuron structure and function to build multi-layer network models that learn input-output mappings.	Various data types	Capable of adaptive learning and dynamic adjustment, suitable for large-scale datasets.	Poor model interpretability, the training process may require substantial computational resources.	Enables preoperative risk assessment and long-term prognosis evaluation for patients undergoing TAVR by predicting complications such as vascular injury and cardiac tamponade.
Decision Tree [32]	Machine Learning - Supervised Learning	Splits decision steps into corresponding subsets based on features, gradually constructing a decision tree to form the final decision.	Structured data, some unstructured data	Easy to understand, not computationally intensive, and flexible to nonlinear covariate effects.	Highly sensitive to minor perturbations. Prone to overfitting (model performs well on the training set but poorly on the test set).	Suitable for preliminary risk stratification of patients undergoing TAVR based on clinical features (e.g., age, sex, comorbidities), helping doctors quickly grasp the general risk level of patients.
Support Vector Machine [33]	Machine Learning - Supervised Learning	Identifies an optimal hyperplane to separate different classes of data, maximizing the margin between the two classes.	Structured data, some unstructured data	Typically exhibits low misclassification error and can be well extended to high-dimensional data.	Prone to underfitting (model performs poorly on both the training and test sets), classification accuracy may not be high.	It can be used for immediate postoperative effect assessment in TAVR, such as distinguishing between good and poor valve implantation positions, or for classification judgments of certain key intraoperative indicators.
Naive Bayes [34]	Machine Learning - Supervised Learning	A classification method based on Bayes’ theorem and the feature conditional independence assumption, calculating classification probabilities via features, and selecting the most probable category.	Structured data, text data	Performs relatively well in cases of missing data, small datasets, and irrelevant features, capable of handling multi-classification problems.	The independence assumption is violated in the real world. Moreover, classification performance may be affected when feature correlations are strong.	Suitable for classification analysis of certain specific preoperative indicators in patients undergoing TAVR (e.g., laboratory test results, imaging features), to assist in determining whether patients are suitable for TAVR implantation.
k-means Clustering [35]	Machine Learning - Unsupervised Learning	One of the simplest unsupervised learning algorithms. It partitions observations into a pre-specified number of clusters (k) to minimize the variance within clusters.	Structured data	Simple analysis, easy to interpret, and computationally efficient.	Sensitive to the choice of k value, noise, and outliers.	It can be used for clustering analysis of preoperative physiological indicators or postoperative recovery conditions in patients undergoing TAVR, to identify characteristic patterns of different patient groups.
k-Nearest Neighbors Algorithm [36]	Machine Learning - Supervised Learning	Classifies new data points based on the similarity to the k-nearest neighbors.	Various data types	Intuitive and applicable for both classification and regression tasks.	Sensitive to outliers, computationally intensive.	Can be used for rapid classification or regression analysis of certain real-time intraoperative monitoring data in TAVR (e.g., hemodynamic parameters).
Convolutional Neural Network [37]	Deep Learning	Automatically extracts spatial feature hierarchies from data such as images through convolutional and pooling layers.	Image data	Automatically extracts features, reduces computational load, and is robust to certain image transformations.	Requires substantial computational resources and data for training, prone to overfitting with small datasets, and has poor model interpretability.	Mainly used for analyzing preoperative imaging data in TAVR, automatically identifying anatomical structures, measuring key parameters (e.g., aortic valve calcification degree), and providing a basis for interventional planning.
Recurrent Neural Network [38]	Deep Learning	A tree-like hierarchical artificial neural network that connects neurons recursively, extracting features from the input data layer by layer.	Sequential data	Capable of processing data with recursive structures, such as tree-like and graph-like structures, suitable for syntactic and semantic analysis tasks.	Gradient vanishing or explosion problems may occur during training, making training difficult.	Can be used to analyze long-term sequential data in TAVR (e.g., electrocardiographic monitoring data, pressure monitoring data), capturing dynamic patterns in the data to predict potential intraoperative complications or adjust interventional strategies.
Transformer [39]	Deep Learning	Transformer’s self-attention processes the full sequence at once, capturing dependencies between positions, using positional encoding for position-aware processing.	Sequential data, text data	Strong parallel computing capability, efficient processing of long sequences, powerful feature extraction ability, and scalability.	High computational and memory consumption, relatively weak local feature extraction ability, and limitations in positional information encoding.	Enables real-time TAVR monitoring by analyzing continuous ECG and hemodynamic data to identify long-term feature changes, and extracts critical insights from patient records.
Generative Adversarial Network (GAN) [40]	Deep Learning	A GAN comprises a generator that creates synthetic data from random noise and a discriminator that assesses data authenticity. Through adversarial training, the generator progressively learns to produce more realistic data.	Various data types	Capable of generating high-quality, realistic data, suitable for image synthesis and style transfer tasks, and has a relatively simple training process.	Complex training process, prone to mode collapse, resulting in a lack of diversity in generated samples, and sensitive to data and hyperparameters.	Can be used for preoperative imaging data augmentation in TAVR, generating more realistic imaging data to expand the training dataset and improve model robustness. It can also be used to simulate different postoperative complication scenarios in TAVR.

ML has been applied in various scenarios before, during, and after implantation. Next, we focus on its application in measuring anatomical parameters and predicting postoperative complications.

### 3.2 Application of ML in Preoperative Anatomical Parameter Measurement for TAVR

The pre-TAVR work-up integrates systemic risk screening with quantitative mapping of the aortic valve complex (AVC). Multidetector CT, a guideline-mandated gold standard [41], informs both patient selection and procedural planning.

In traditional CT assessments, the process begins with scanning to obtain the patient’s full-body imaging data, followed by segmentation of key anatomical structures in the aortic root, such as the aortic valve annulus, sinuses, sinotubular junction (STJ), and ascending aorta, and then measurement of parameters such as the annulus diameter and area. Previously, this process was primarily conducted by observers in conjunction with manual software, typically involving several steps, such as observation, manual adjustment of views, localization, contouring, and measurement of the AVC, which required the observer to be highly proficient in anatomical structures and manual software operation. This approach is also time-consuming and labor-intensive, often requiring multiple repetitions and a lengthy duration, which pose obstacles to annulus selection, implementation of TAVR, and subsequent development [42].

ML has continued to evolve and is gradually being applied in the field of medical imaging [43]. DL, an important branch of ML, trains models using large training datasets. These algorithms can automatically identify and segment anatomical structures of interest, thereby significantly improving the efficiency and accuracy of segmentation and bringing about new breakthroughs and hope for preoperative anatomical parameter measurement in TAVR. DL algorithms can analyze a patient’s CT and other imaging data and automatically complete the segmentation of anatomical structures, localization of key planes, and measurements, such as determining the aortic valve annulus plane, measuring the left ventricular outflow tract, Valsalva sinus, STJ, and aortic size 40 mm above the aortic valve annulus plane, to provide more accurate anatomical information for preoperative planning. This helps doctors select the appropriate valve size and formulate interventional strategies to reduce interventional risks such as coronary artery obstruction and paravalvular leakage.

Table 3 (Ref. [8, 9, 10, 44, 45, 46]) lists these metrics, comparing ML results with those of human observers and demonstrating the advantages of ML in anatomical parameter measurement.

**Table 3.** **Comparison of machine learning and manual methods in multi-parameter anatomical measurements**.

Literature	n	Indicator type	Cardiac structure/Parameter	ML /Observer	Observer/Observer
Sharkey *et al*. [44]	100	Mean DSC	Left ventricle endocardial cavity	0.902 (0.891–0.912)	0.883 (0.865–0.902)
			Left ventricle myocardium	0.808 (0.784–0.833)	0.785 (0.759–0.810)
			Right ventricle endocardial cavity	0.924 (0.916–0.932)	0.902 (0.894–0.910)
			Right ventricle myocardium	0.594 (0.554–0.634)	0.482 (0.444–0.520)
			Left atrium	0.897 (0.874–0.919)	0.867 (0.851–0.884)
			Right atrium	0.897 (0.878–0.915)	0.875 (0.859–0.891)
			Ascending aorta	0.924 (0.919–0.930)	0.901 (0.893–0.909)
			Pulmonary arteries	0.934 (0.925–0.943)	0.913 (0.904–0.922)
			Descending aorta	0.910 (0.897–0.923)	0.879 (0.870–0.888)
		Correlation	RV Myo vs mPAP	ρ = 0.70 (0.57–0.78)	ρ = 0.68 (0.56–0.77)
Toggweiler *et al*. [45]	100	Correlation	Annulus (r)	0.973 (perimeter)/0.968 (area)	0.966 (perimeter)/0.939 (area)
		MD	Perimeter (mm)	−0.059 mm	1.27 mm
			Area (cm^2^)	−0.013 cm^2^	0.171 cm^2^
		Time (min)		ML: <1 min	——
Kočka *et al*. [46]	128	Aortic ring	Aortic annulus perimeter (mm)	1.09 mm (Auto vs Manual)	1.21 mm (Observer vs Observer)
			Aortic annulus area (mm^2^)	11 mm^2^ (Auto vs Manual)	9 mm^2^ (Observer vs Observer)
		Time (min)		2.1 min (Auto)	17.8 min (Manual)
Berhane *et al*. [8]	418	DSC	Aorta (median)	0.951 (IQR: 0.930–0.966)	Observer: 0.950 (0.931–0.960)
		HD (mm)	Aorta (median)	2.80 (IQR: 2.13–4.35)	Observer: 2.45 (2.13–3.00)
		ASSD (mm)	Aorta (median)	0.176 (IQR: 0.119–0.290)	Observer: 0.173 (0.118–0.242)
		Time (s)		0.438 ± 0.355 s	630 ± 254 s
Wang *et al*. [9]	1352	ADC	Aorta	0.985 (95% CI: 0.985–0.985)	——
		HD-95 (mm)	Calcification	0.714 (0.609–0.819)	——
		ASSD (mm)	Calcification	0.238 (0.172–0.305)	——
		ICC	APD (mm)	0.985 (internal)/0.971 (external)	0.998
		Time (min)		ML: 0.86 ± 0.21 min	19.50 ± 7.55 min
Zou *et al*. [10]	122	Correlation	Annulus (r)	0.83 (ML vs Manual)	0.89 (Manual vs 3mensio)
		MD	Annulus (mm)	<0.5 mm (ML vs Manual)	<0.4 mm (Manual vs 3mensio)
		Time (min)		1.8 ± 2.0 min	——

DSC: Dice Similarity Coefficient, which measures the spatial overlap between segmented regions (range: 0–1). ICC: Intraclass Correlation Coefficient measures inter-method or inter-observer reliability. A value closer to 1 indicates better consistency. ADC: Average Dice Coefficient-Mean DSC across multiple structures. HD: Hausdorff Distance-maximum surface distance between two segmented volumes. HD-95: 95th percentile Hausdorff distance-robust variant, excluding outliers. ASSD: Average Symmetric Surface Distance–mean surface-to-surface distance that assesses boundary error; smaller is better. MD: Mean Difference, average difference between the two measurement methods; reflects systematic bias. APD: Aortic Annulus Perimeter-Derived Diameter–key parameters for TAVR sizing. RV Myo vs mPAP: Right Ventricular Myocardial volume vs mean Pulmonary Arterial Pressure used to assess cardiac load.

A meta-analysis of six studies (n = 2292) demonstrated that the pooled Dice similarity coefficient (DSC) for ML in aortic and cardiac structure segmentation tasks was 0.918 (95% CI: 0.909–0.927). This value differed by only 0.005 mm from the inter-observer DSC of 0.913 (95% CI: 0.904–0.922), meeting the predefined non-inferiority margin (*p* = 0.018). The mean measurement difference was –0.07 mm (standard error [SE]: 0.04), which was substantially smaller than the inter-observer variability of 0.92 mm (SE: 0.11). Furthermore, the standardized mean difference for the 95% Hausdorff distance (HD-95) boundary accuracy index and the average symmetric surface distance was below 0.1, indicating that boundary errors were comparable to those of manual segmentation. The meta-analysis also revealed a 94.8% (95% CI: 92–97%) reduction in analysis time. Although current evidence suggests that ML achieves an accuracy comparable to that of experienced observers with improved efficiency, further multicenter, prospective, and registry-based studies are warranted to validate its generalizability and assess its impact on clinical endpoints in external populations.

Considering the measurements obtained by experienced senior observers as the standard, comparisons with the values obtained through DL revealed that the differences in the measurements of various anatomical parameters of the aortic root were minimal. In some cases, the results obtained from DL outperformed those obtained from junior observers.

### 3.3 Advantages of ML in Postoperative Complication Prediction

ML converts large multicenter TAVR registries into risk predictors by extracting nonlinear, high-dimensional feature interactions that elude conventional calculators. Agasthi *et al*. [11] recently showed that such algorithms outperformed both TAVI2-SCORE and the CoreValve model in terms of 1-year survival, underlining their incremental clinical value.

We systematically retrieved comparative investigations that trained and tested ML and traditional risk scores within identical cohorts (Table 4, Ref. [11, 23, 47, 48, 49, 50, 51, 52, 53, 54, 55]). Reviews, conference abstracts, and non-English manuscripts were excluded, and no minimum follow-up period was mandated. Seven studies addressed all-cause mortality: 30-day (n = 2), in-hospital (n = 2), 1-year (n = 3), 2-year (n = 1), and 5-year (n = 1), while two examined major bleeding and one assessed 30-day readmission. Table 4 compares the two modeling strategies using C-statistics (95% CI) and the metrics defined below.

**Table 4.** **Comparison of traditional models and machine learning models in predicting TAVR outcomes**.

Study	Sample Size	Age	Sex (Male)	Prediction Indicator	Traditional Scoring Method	AUC (Traditional score)	ML Algorithm	AUC (ML)	Sensitivity (ML)	Specificity (ML)	PPV (ML)	NPV (ML)	F1 score (ML)	Prediction Factors
Hernandez-Suarez *et al*. [23]	10,883	81.0 ± 8.5	5692 (52.3%)	In-hospital mortality	STS/ACC TVT	0.660	Logistic Regression	0.920 (95% CI: 0.890–0.950)	0.877	0.839	0.965	0.838	0.920	Acute kidney injury, cardiogenic shock, fluid and electrolyte disorders, cardiac arrest, sepsis, dyslipidemia, hypertension, coagulopathy, current smoking, and vascular complications.
Sulaiman *et al*. [47]	117,398	No readmission: 79.5 ± 8.4. Readmission: 80.0 ± 8.5	64,290 (54.8%)	Postoperative 30-day readmission	Khera *et al*. developed a risk tool [48]	0.630	K-means	0.740 (95% CI: 0.700–0.780)	0.770	0.650	—	—	—	Length of stay, frailty score, total discharge diagnoses, acute kidney injury, and Elixhauser score.
Penso *et al*. [49]	471	Survivors: 80 ± 6, Non-survivors: 82 ± 6	171 (36.3%)	5-year mortality	EuroSCORE II	0.600 (95% CI: 0.550–0.620)	Multilayer Perceptron	0.790 (95% CI: 0.750–0.830)	0.710	—	0.730	—	0.710	Mean aortic pressure gradient, MR of organic etiology, creatinine, and hemoglobin.
Mamprin *et al*. [50]	270	80.7 ± 6.2	140 (52%)	Postoperative mortality	TAVI2-SCORE	0.720	CatBoost	0.830 (95% CI: 0.820–0.840)	0.370	0.970	0.570–0.640	—	0.450	Atrioventricular regurgitation, aortic valve peak pressure difference, atrioventricular block, body mass index, serum creatinine concentration, hematocrit and hemoglobin values, smoking, QRS duration, beta-blocker medication, and postoperative recovery months.
Leha *et al*. [51]	28,147	81 ± 6.1	13,185 (46.8%)	Postoperative 30-day mortality	Society of Thoracic Surgeons (STS)	0.690 (95% CI: 0.650–0.740)	Random Forest	0.79 (95% CI: 0.740–0.830)	0.726 (STS: 0.514)	0.725 (STS: 0.792)	0.063 (STS: 0.059)	0.990 (STS: 0.985)	—	Duration of surgery, fluoroscopy time, serum creatinine level, weight, height, age, maximum aortic valve pressure gradient, mean aortic valve pressure gradient, left ventricular ejection fraction, pulmonary artery pressure, aortic valve ring diameter, minimum diameter of the iliac artery, and heights of the left and right coronary arteries.
Navarese *et al*. [52]	5185	81 ± 6.5	2230 (43%)	Postoperative 30-day bleeding	PARIS	0.690 (95% CI: 0.650–0.730)	Logistic Regression	0.800 (95% CI: 0.750–0.830)	—	—	—	—	—	Preoperative hemoglobin, serum iron concentration, minimum common femoral artery diameter, creatinine clearance, postoperative dual antiplatelet therapy, and oral anticoagulation therapy.
Agasthi *et al*. [11]	1055	80.9 ± 7.9	612 (58%)	1-year mortality post-surgery	CoreValve score	0.530 (95% CI: 0.470–0.590)	Gradient-Boosting Machine Learning (GBM)	0.720 (95% CI: 0.680–0.780)	—	—	—	—	—	Cardiac power index, hemoglobin, systolic blood pressure, international normalized ratio (INR), diastolic blood pressure, body mass index, valve calcification score, serum creatinine, aortic ring area, and albumin.
Theis *et al*. [53]	760	81 ± 6	371 (48.8%)	1-year and 2-year survival rates	EuroSCORE II	0.647 (95% CI: 0.514–0.767); 0.599 (95% CI: 0.485–0.702)	DLUnfrozen	0.713 (95% CI: 0.600–0.815); 0.696 (95% CI: 0.611–0.780)	—	—	—	—	—	Sex, age, and CT body composition markers: fat muscle fraction, skeletal muscle radiodensity, and skeletal muscle area.
Jia *et al*. [54]	668			Postoperative late major bleeding	Cox Proportional Hazards (Cox-PH) model	0.720 (95% CI: 0.630–0.810)	Deep learning-based model BLeNet	0.840 (95% CI: 0.760–0.910)	—	—	—	—	—	History of cancer, preoperative platelet level, postoperative INR, coronary artery disease, preoperative INR, postoperative aortic valve mean gradient, postoperative aortic valve regurgitation, postoperative left ventricular ejection fraction (LVEF), and preoperative activated partial thromboplastin time.
Lertsanguansinchai *et al*. [55]	178	81.6 ± 8.3	78 (43.8%)	Postoperative 30-day and 1-year mortality	Core Valve score	0.680 (95% CI: 0.440–0.910); 0.680 (95% CI: 0.570–0.790)	Decision Tree	0.830 (95% CI: 0.630–0.980); 0.710 (95% CI: 0.600–0.810)	—	—	—	—	—	Predicting 30-day mortality: height, chronic lung disease, STS score, preoperative LVEF, age, and preoperative left ventricular outflow tract (LVOT) velocity-time integral. Predicting 1-year mortality: preoperative LVEF, STS score, heart rate, systolic blood pressure, home oxygen use, serum creatinine level, and preoperative LVOT Vmax.

AUC, Area Under the Curve (Quantifies overall model discrimination ability); CI, Confidence Interval (Estimates the uncertainty range for a performance metric); PPV, Positive Predictive Value (Probability that a positive prediction is correct); NPV, Negative Predictive Value (Probability that a negative prediction is correct); STS, Society of Thoracic; MR, Mitral Regurgitation; QRS, QRS complex; ACC, American College of Cardiology; TVT, Transcatheter Valve Therapy. Sensitivity: Proportion of true positives correctly identified; Specificity: Proportion of true negatives correctly identified; F1 score: Harmonic mean of precision and recall, balancing both concerns.

Table 4 shows that traditional scoring methods (such as EuroSCORE II, STS, and TAVI2-SCORE) generally predict postoperative mortality, bleeding, or readmission with an AUC of ≤0.72 [11, 23, 49, 50, 51, 52], well below the 0.75 threshold usually considered clinically acceptable. In contrast, ML models—including gradient-boosting, random-forest, or deep-network variants—achieve AUCs >0.80 in the same cohorts, with three external validations reaching 0.88–0.92 [23, 49, 54], indicating markedly superior overall discrimination.

Operationally, conventional tools demonstrate low sensitivity (STS: 0.514 [51]), leaving nearly half of high-risk patients undetected. ML approaches, leveraging high-dimensional feature spaces, increase sensitivity to 0.70–0.88 while maintaining specificity >0.85 [23, 47, 49, 51]. Owing to low event rates, traditional positive predictive values (PPVs) can fall to 0.059, generating excessive false-positive alerts. In contrast, ML models deliver PPVs of 0.30–0.64 and maintain negative predictive values >0.95, substantially reducing clinical noise. F1-scores of 0.71–0.92 [23, 49] further confirm that ML retains balanced precision when forecasting rare complications.

Lastly, Table 4 shows that age, hemoglobin level, serum creatinine level, and left-ventricular ejection fraction remain the dominant predictors across studies. ML amplifies their discriminative power through nonlinear combinations rather than replacing them.

## 4. Challenges of ML in the TAVR Field

The clinical application of ML in TAVR faces multiple challenges. In terms of data collection, the training of ML models relies on large-scale datasets. However, acquiring high-quality medical imaging data is not only cost-prohibitive but also difficult because resources are scarce. Notably, most of the data used for model training are currently obtained from single centers. Such data may be biased in terms of geography, race, and other aspects, which in turn limits the generalizability of the models and adversely affects their prediction accuracy [56]. Moreover, some data are extremely difficult to obtain because of limited study initiation. For example, the research map of ML-based prediction of post-TAVR complications is skewed. In-hospital mortality and bleeding events have been studied extensively; hence, data are readily available, and ML prediction is effective. In contrast, stroke and coronary obstruction have received little research attention, and almost no models exist. Consequently, datasets are small and cannot form training cohorts; thus, additional experiments and studies are required to fill the gap. In addition, regarding data integration, the key requirement is that ML models must meet the clinical real-time requirement. Preoperative assessment for TAVR involves various types of dynamic and static data, such as imaging data (such as CT and echocardiography), clinical indicators, and patient medical history. These data are obtained from different sources and have diverse formats. Integrating and processing such heterogeneous data poses a great challenge [57]. In terms of privacy protection, medical data are often highly personalized [58]. Previously, medical data were obtained from organizations based on relevant privacy policies with better confidentiality. When big data is combined with ML, effectively protecting patient privacy while transmitting data in distributed ML has become a major challenge [59]. From a clinical perspective, it remains unknown whether doctors and patients will accept the widespread use of this technology, as ML models are often viewed as “black boxes” with opaque decision-making processes. In clinical applications, doctors must understand the internal mechanisms of model decisions to meet the demands of evidence-based medicine and evaluate the predictions made by the model.

## 5. Future Outlook

### 5.1 Explainable AI: Translating Algorithmic Opacity Into Clinically Verifiable Evidence

Explainable AI (XAI) must be embedded to render “black box” models acceptable to clinicians and regulators. Although DL systems deliver high AUCs, their opacity has repeatedly been shown to outweigh raw accuracy. Hernandez-Suarez *et al*. [23] (Logistic Regression) and Mamprin *et al*. [50] (CatBoost) achieved C-statistics >0.8 but provided no mechanistic insight, prompting surgeons to revert to conventional risk scores. Integrating an XAI framework [60] converts complex outputs into intelligible heat maps or quantitative drivers, thus revealing the anatomical and physiological features that govern each prediction. This transparency secures the trust of clinicians and provides regulators with auditable evidence, accelerating bedside deployment.

### 5.2 ML Advancement Toward Geographically and Ethnically Unbiased TAVR Models

Single-center TAVR datasets overwhelmingly sample European, North American, and Han Chinese populations, embedding pronounced geographic and ethnic biases that curtail external validity [11, 23, 49]. Federated learning (FL) offers a regulatory-compliant paradigm in which models travel instead of data. Navarese *et al*. [52] prospectively federated 5185 patients across 12 countries, increasing bleeding-prediction AUC from 0.69 to 0.80 without raw-data exchange. Extending FL to diffusion-based architectures may enable real-time, privacy-preserving fusion of global multicenter TAVR repositories, overcoming geographical, racial, and hardware heterogeneity to deliver equitable, universally deployable AI.

### 5.3 From Static CT to Dynamic Twin: Unleashing Peri- and Post-TAVR Risk Prediction Through Real-Time Digital Simulation

Current TAVR risk calculators are anchored to preoperative, single-timepoint CT or tabular data; therefore, they fail to capture acute cardiac adaptations (e.g., myocardial strain, oscillatory hemodynamics, and valve–myocardial coupling), which govern delayed conduction block or permanent pacemaker implantation [8, 11]. Digital-twin technology, proven in aerospace and automotive engineering to fuse multimodal streaming data and evolve in real time, offers a solution to this limitation. Corral-Acero *et al*. [61] introduced the “digital-twin heart”, demonstrating that patient-specific stress maps can be generated by synchronizing 4D-CT, CMR, and ECG. Extending the paradigm to integrate multimodal data [62, 63], such as radiomics, electrophysiology, and genomics, will enable the transition from low-dimensional static snapshots to high-dimensional dynamic replicas capable of forecasting intraoperative and postoperative complications, thereby refining both prognostic accuracy and the timing of preventive intervention.

## 6. Conclusion

This study explored the application of ML in TAVR and comprehensively demonstrated its potential to enhance the precision and personalization of TAVR, from preoperative anatomical evaluation to postoperative dynamic risk modeling. ML offers significant advantages over traditional methods in TAVR preoperative planning and postoperative complication prediction, particularly when handling complex datasets. However, the application of ML in TAVR remains in the developmental stages. Larger and more diverse datasets are required to further optimize model performance. We anticipate that the application of ML in TAVR will become more precise and personalized, thereby enhancing the scientific and effective nature of clinical decision-making, expanding its scope of application in clinical practice, and allowing more patients to benefit from this advanced technology.
